# Elevated CO_2_ Enhances Dynamic Photosynthesis in Rice and Wheat

**DOI:** 10.3389/fpls.2021.727374

**Published:** 2021-10-01

**Authors:** Huixing Kang, Ting Zhu, Yan Zhang, Xinran Ke, Wenjuan Sun, Zhenghua Hu, Xinguang Zhu, Haihua Shen, Yao Huang, Yanhong Tang

**Affiliations:** ^1^Key Laboratory for Earth Surface Processes of Ministry of Education, Department of Ecology, College of Urban and Environmental Sciences, Peking University, Beijing, China; ^2^State Key Laboratory of Vegetation and Environmental Change, Institute of Botany, Chinese Academy of Sciences, Beijing, China; ^3^School of Applied Meteorology, Nanjing University of Information Science and Technology, Nanjing, China; ^4^Center of Excellence for Molecular Plant Sciences, State Key Laboratory of Plant Molecular Genetics, Chinese Academy of Sciences, Shanghai, China

**Keywords:** acclimation, dynamic photosynthesis, elevated CO_2_, photosynthetic induction, rice, wheat

## Abstract

Crops developed under elevated carbon dioxide (eCO_2_) exhibit enhanced leaf photosynthesis under steady states. However, little is known about the effect of eCO_2_ on dynamic photosynthesis and the relative contribution of the short-term (substrate) and long-term (acclimation) effects of eCO_2_. We grew an *Oryza sativa japonica* cultivar and a *Triticum aestivum* cultivar under 400 μmol CO_2_ mol^−1^ air (ambient, *A*) and 600 μmol CO_2_ mol^−1^ air (elevated, *E*). Regardless of growth [CO_2_], the photosynthetic responses to the sudden increase and decrease in light intensity were characterized under 400 (*a*) or 600 μmol CO_2_ mol^−1^ air (*e*). The *Aa*^1^, *Ae*^2^, *Ea*^3^, and *Ee*^4^ treatments were employed to quantify the acclimation effect (*Ae* vs. *Ee* and *Aa* vs. *Ea*) and substrate effect (*Aa* vs. *Ae* and *Ea* vs. *Ee*). In comparison with the *Aa* treatment, both the steady-state photosynthetic rate (*P*_N_) and induction state (IS) were higher under the *Ae* and *Ee* treatments but lower under the *Ea* treatment in both species. However, IS reached at the 60 sec after the increase in light intensity, the time required for photosynthetic induction, and induction efficiency under *Ae* and *Ee* treatment did not differ significantly from those under *Aa* treatment. The substrate effect increased the accumulative carbon gain (ACG) during photosynthetic induction by 45.5% in rice and by 39.3% in wheat, whereas the acclimation effect decreased the ACG by 18.3% in rice but increased it by 7.5% in wheat. Thus, eCO_2_, either during growth or at measurement, enhances the dynamic photosynthetic carbon gain in both crop species. This indicates that photosynthetic carbon loss due to an induction limitation may be reduced in the future, under a high-CO_2_ world.

## Introduction

According to IPCC (2014), “the atmospheric CO_2_ concentration is projected to reach beyond 550 μmol CO_2_ mol^−1^ air by 2100 under high emissions scenarios.” increase in atmospheric CO_2_ has been reported to enhance photosynthesis under constant light conditions, i.e., steady-state photosynthesis (Ainsworth and Long, 2005; Ainsworth and Rogers, 2007). However, terrestrial plants in natural environments are exposed to fluctuating light (Pearcy, 1983; Tang et al., 1988; Pearcy et al., 1990), which increases leaf carbon gain more than steady light under long-term exposure to elevated CO_2_ (eCO_2_) (Leakey et al., 2002). Thus, knowledge about photosynthetic responses to fluctuating light, i.e., dynamic photosynthesis, at eCO_2_ conditions helps improve our understanding of the future global carbon flux.

Plasticity in photosynthesis in response to long-term eCO_2_ can be attributed to the short-term (substrate) effect, long-term (acclimation) effect, or both. The substrate effect is related to increased CO_2_ supply, which is reported to enhance dynamic photosynthesis greatly (Tomimatsu and Tang, 2012; Tomimatsu et al., 2014, 2019; Kaiser et al., 2017b). The acclimation effect is related to variations in leaf morphological, anatomical, and biochemical traits, but little information is known about its role and relative contribution.

The activation of Calvin-Benson cycle enzymes and stomatal opening regulate the photosynthetic response to a sudden increase in light intensity (Way and Pearcy, 2012; Kaiser et al., 2015) and are affected by variations in leaf chemical and morphological traits. Leaves exposed to long-term eCO_2_ have a lower Rubisco activase (Rca) content (Geiger et al., 1999; Aranjuelo et al., 2011; Tomimatsu et al., 2019) and develop small stomata (Maherali et al., 2002; Zhu et al., 2018; Zheng et al., 2019). The rate of Rubisco activation is proportional to Rca content (Woodrow and Mott, 1989; Yamori et al., 2012). Stomatal morphology affects the stomatal opening kinetics in some species (Drake et al., 2013; Kardiman and Ræbild, 2018; Zhang et al., 2019), although contradictory evidence has also been reported (Elliott-Kingston et al., 2016; McAusland et al., 2016). Whether and how acclimation to eCO_2_ affects dynamic photosynthesis remains untested.

All studies listed in Table 1 have investigated woody species, except for one study on C_4_ grass (Knapp et al., 1994). Rice and wheat are important for the global population as direct sources of food (FAO, 2016); yet, there are no available reports on the effect of long-term eCO_2_ on dynamic photosynthesis in rice, wheat, or any other C_3_ crop plants. The light environments for crop plants are characterized by long periods of sunlight punctuated by shadeflecks (Pearcy et al., 1990), whereas those for within-canopy woody plants are characterized by long periods of diffuse light punctuated by sunflecks (Tang et al., 1988; Chazdon and Pearcy, 1991). The slow photosynthetic induction in wheat costs at least 21% of its daily potential assimilation (Taylor and Long, 2017). Thus, it is of great importance to investigate whether acclimation to eCO_2_ improves dynamic photosynthesis in C_3_ crop plants.

**Table 1:** Summaries of previous studies addressing the effect of long-term elevated carbon dioxide (eCO_2_) on photosynthetic induction.

**References**	**Species**	**Metabolism/**	**CO_**2**_ treatment**	**Treatment**	**Percentage change per 100** **μmol CO**_**2**_ **mol**^**−1**^ **air (%)**				
		**life form**	**(μmol CO_**2**_ mol^**−1**^ air)**	**duration**					
					***T_***P***_*_**50%**_**	***T_***P*9**_*_**0%**_**	***T_***g***_*_**50%**_**	***T_***g***_*_**90%**_**	**τ_R_**
Knapp et al. (1994)	*Andropogon gerardii* Vitman	C_4_/grass	ambient vs. double ambient^†^	<1 year					
Naumburg and Ellsworth (2000)	*Acer rubrum*	C_3_/woody	365 vs. 569	14–15 years	U	U			
	*Liriodendron tulipifera*	C_3_/woody	365 vs. 569	14–15 years	U	U			
	*Cornus florida*	C_3_/woody	365 vs. 569	14–15 years	U	U			
	*Liquidambar styraciflua*	C_3_/woody	365 vs. 569	14–15 years	U	U			
Leakey et al. (2002)	*Shorea leprosula* Miq.	C_3_/woody	377 vs. 710	216 days	**+29.71**	**−8.00**	**+14.00**	**+7.43**	
Holišova et al. (2012)	*Fagus sylvatica*	C_3_/woody	355 vs. 724	3 year		−1.43			
	*Picea abies*	C_3_/woody	355 vs. 724	3 year		**−8.00**			
Tomimatsu and Tang (2012)	*Populus euramericana* cv. I-55	C_3_/woody	380 vs. 720	60 days	**−24.61**	**−15.62**			
	*Populus koreana × trichocarpa* cv. Pea	C_3_/woody	380 vs. 720	60 days	**−11.14**	−5.01			
Tomimatsu et al. (2019)	*Populus koreana × trichocarpa* cv. Pea	C_3_/woody	380 vs. 1020	60 days	**−7.16**	**−3.14**			

*Data were taken from tables and figures shown in the articles using WebPlotDigitizer version 4.2 (https://automeris.io/WebPlotDigitizer/index.html). Shown are the normalized effects of eCO_2_, which were calculated as percentage changes in parameters divided by the differences in CO_2_ concentrations applied. “U” indicates no changes in the parameter. Numbers in bold style indicate significant eCO_2_ effects on the parameters at P = 0.05 level*.

† *Ambient CO_2_ concentration was 330–340 ml L^−1^*.

In this study, we aimed to address: (1) how eCO_2_ affects dynamic photosynthesis; (2) the relative contribution of the acclimation and substrate effects of eCO_2_ on dynamic photosynthesis.

## Materials and Methods

### Plant Materials and Growth Conditions

The study was conducted at the Agricultural Meteorology and Ecology Experimental Station, Nanjing University of Information Engineering, Nanjing, China (32°16′N, 118°46′E). An *Oryza sativa japonica* cultivar (Nanjing 9108) and a *Triticum aestivum* cultivar (Yangmai 22) were used. There are many cultivars of rice and wheat since both are widely cultivated crop species with a very long cultivation history. Nanjing 9108 was nominated as “super rice” by the Chinese Ministry of Agriculture in 2015, whereas Yangmai 22 is a high-yield wheat cultivar. Currently, both are the major and most widely cultivated cultivars in the middle and lower reaches of the Yangtze River. The rice seeds were sown (rice, May 2017; wheat, October 2017) at 400 μmol CO_2_ mol^−1^ air. Afterward, the rice seedlings were transplanted into octagonal open-top chambers (OTCs) with 400 (ambient, denoted by *A*) or 600 μmol CO_2_ mol^−1^ air (elevated, denoted by *E*). The wheat seeds were directly sown in the soil in each OTC. The OTCs were 3 m high with a bottom area of ~12 m^2^. Ventilation fans were installed on the inner walls of the OTCs to minimize the impact of heterogeneous temperature and CO_2_ concentration on the height of the flag leaves. The plants grown within the core area of an ambient [CO_2_] OTC and an elevated [CO_2_] OTC were selected for the experiment. The soil was carefully plowed before transplanting the rice seedlings and sowing the wheat seeds. These practices allowed for fairly homogeneous soil conditions for the two CO_2_ treatments (OTCs), which were only 8–10 m apart from each other. The soil nutrition content at a 0–20-cm depth before sowing the wheat seeds was 1.25 vs. 1.34 g N kg^−1^, .84 vs. .83 g P kg^−1^, and 18.1 vs. 18.08 g K kg^−1^ for the ambient vs. elevated OTC, respectively. Atmospheric environments during the growth period were also similar between the two OTCs (Supplementary Figure 1). Furthermore, all plants in the two treatments received the same management practices, e.g., fertilization, irrigation, and pest control. All these conditions ensured that the major difference between the two treatments was growth CO_2_ concentration. We then focused on the biological replication, i.e., the individual plants, for photosynthetic measurement replicates. The measurements were conducted during grain filling: rice, September 15–28, 2017; wheat, April 20–27, 2018.

### Gas Exchange Measurement

In both species, flag leaves contribute an important portion of assimilates used for grain filling (Yoshida, 1981; Carmo-Silva et al., 2017). We then decided on all the measurements to be conducted with the flag leaves. Gas exchange parameters were measured on the south-facing, fully expanded flag leaves with portable infrared gas analyzers (Li-Cor 6400 and Li-Cor 6800, LI-COR Biosciences, Lincoln, NE, USA). Air temperature during grain filling was slightly higher for rice than for wheat; therefore, the block temperature was set to 32.5°C for rice and 30°C for wheat. Relative humidity was maintained at 60–65%. Measurements were made on three to four leaves from different plants (one leaf per plant).

To determine the responses of the net photosynthetic rate to the intercellular CO_2_ concentration (*P*_N_-*C*_i_ curves, equivalent to *A*-*C*_i_ curves as in common usage), leaves were acclimated to a saturating light intensity of 1,500 μmol photons m^−2^ s^−1^ at a CO_2_ concentration identical to growth [CO_2_]. Then, the CO_2_ concentration in the reference cell was varied from 50 to 1,500 μmol CO_2_ mol^−1^ air. Altogether, 14 different CO_2_ concentrations were investigated.

To characterize dynamic photosynthesis, we measured the photosynthetic time course in response to a simulated increase and decrease in light intensity. The leaves were first acclimated at 100 μmol photons m^−2^ s^−1^ for over 30 min, then the light intensity within the leaf chamber was increased to 1,500 μmol photons m^−2^ s^−1^, and kept constant for 5 min. Afterward, the light intensity was decreased to 50 μmol photons m^−2^ s^−1^ and kept constant for 5 min. The net photosynthetic rate and stomatal conductance (*g*_s_) were recorded every second for the entire measurement period. Photosynthetic characteristics in plants grown at 600 μmol CO_2_ mol^−1^ air were influenced not only by the short-term but also the long-term effect of eCO_2_, in comparison with plants grown at 400 μmol CO_2_ mol^−1^ air. To distinguish both effects, we measured dynamic photosynthesis at 400 (denoted by *a*) and 600 μmol CO_2_ mol^−1^ air (denoted by *e*), regardless of growth [CO_2_]. The differences between the two growth conditions [CO_2_] under the same measurement [CO_2_] (i.e., *Ae* vs. *Ee* and *Aa* vs. *Ea*) were considered to be caused by the acclimation effect of eCO_2_, while the differences between the two measurements [CO_2_] from the same growth [CO_2_] (i.e., *Aa* vs. *Ae* and *Ea* vs. *Ee*) were considered to be due to the instantaneous substrate effect of eCO_2_.

### Carbon and Nitrogen Analysis

The leaves used for the gas exchange measurements were collected, brought to the laboratory, and then oven-dried at 65°C for 48 h before grinding. The carbon and nitrogen concentrations of ground samples were determined using a CHNOS elemental analyzer (Vario EL III, Elementar Analysensysteme GmbH, Langenselbold, Germany).

### Stomatal Anatomy

Five flag leaves other than those used for the gas exchange measurements from the same OTC were sampled and kept in a 2.5% glutaraldehyde solution. Images of the center of the abaxial leaf surface at ×800 magnification for rice and ×400 for wheat were acquired with a scanning electron microscope (FEI Quanta 200F, Thermo Fisher Scientific, Waltham, MA, USA). Stomatal density and guard cell length were determined using the ImageJ software (National Institute of Health, Bethesda, MD, USA) from 10–20 fields of view.

### Data Analysis

To evaluate the rates of photosynthetic and stomatal induction responses, we calculated the time to reach 50 and 90% of the steady-state photosynthetic rate (*P*_N_) (*T*_*P*__50%_ and *T*_*P*__90%_) and *g*_s_ (*T*_*g*__50%_ and *T*_*g*__90%_) at 1,500 μmol photons m^−2^ s^−1^. Photosynthetic induction state (IS) was calculated after Chazdon and Pearcy (1986):


(1) $$IS\left(t\right)=\frac{P_{N}(t)-P_{N,100}}{P_{N,1500}-P_{N,100}}*100\%$$

where *P*_N, 100_ is the steady-state *P*_N_ reached at 100 μmol photons m^−2^ s^−1^ and *P*_N, 1500_ is the steady-state *P*_N_ reached at 1,500 μmol photons m^−2^ s^−1^. Both were calculated by averaging single values over the last half-minute of each period.

We assumed that the transition between the Rubisco-limited state and the ribulose 1,5-bisphosphate (RuBP) regeneration-limited state occurs at a *C*_i_ of 200–400 μmol CO_2_ mol^−1^ air at ambient growth [CO_2_] and a *C*_i_ of 400–600 μmol CO_2_ mol^−1^ air at elevated growth [CO_2_] during the induction response. Then, *P*_N_-*C*_i_ curves were divided into two parts and fitted separately to obtain the apparent maximum Rubisco carboxylation rate (*V*_c, max_), electron transport rate at 1,500 μmol photons m^−2^ s^−1^ (*J*_1500_), and CO_2_ photo compensation point (Γ^*^). Parameters estimated from the *P*_N_-*C*_i_ curves were then used to correct transient *P*_N_ by removing stomatal limitations. Since RuBP regeneration limitation typically relaxes within 2 min (Taylor and Long, 2017) and triose phosphate use limitation barely occurs at the CO_2_ concentration investigated in this study (Long and Bernacchi, 2003), the *P*_N_ was corrected only 2 min after the increase in light intensity. Under Rubisco limitation, *P*_N_ at time *t* can be calculated after Farquhar et al. (1980):


(2) $$P_{N}(t)=V_{c,max}(t)[\frac{C_{i}\left(t\right)-\Gamma^{*}}{C_{i}\left(t\right)+K_{m}}]-R_{L}$$

where *V*_c, max_(*t*) and *C*_i_(*t*) is *V*_c, max_, and *C*_i_ at time *t*, respectively, *K*_m_ is the Michaelis–Menten constants of Rubisco taken from Bernacchi et al. (2001), and *R*_L_ is mitochondrial respiration rate in the light and assumed to be 60% of dark respiration rate (Way et al., 2019), which was determined in a preliminary test. To remove the stomatal limitation, *P*_N_ was recalculated by replacing *C*_i_(*t*) with the final, steady-state *C*_i_ (*C*_i,f_):


(3) $$P_{N}^{*}\left(t\right)=V_{c,max}(t)[\frac{C_{i,f}-\Gamma^{*}}{C_{i,f}+K_{m}}]-R_{L}$$

where $P_{N}^{*}\left(t\right)\;$is corrected *P*_N_(*t*). Combining Equations (1, 2) to eliminate the unknown variable *V*_*c,max*_(*t*):


(4) $$P_{N}^{*}\left(t\right)=\;\frac{\left[P_{N}(t)+R_{L}](C_{i,f}-\Gamma^{*})[C_{i}\left(t\right)+K_{m}\right]}{\left[C_{i}\left(t\right)-\Gamma^{*}](C_{i,f}+K_{m}\right)}-R_{L}$$

The time courses of $P_{N}^{*}$ were then fitted to the model proposed by Woodrow and Mott (1989):


(5) $$P_{N}^{*}\left(t\right)=P_{N,f}^{*}-\left(P_{N,f}^{*}-P_{N,i}^{*}\right)*e^{\left(-\frac{t}{\tau_{R}}\right)}$$

where $P_{N,f}^{*}$ and $P_{N,i}^{*}$ are the final corrected and estimated initial photosynthetic rate, respectively; τ_R_ is the apparent time constant of Rubisco activation. Diffusional and biochemical limitations were estimated after Kaiser et al. (2017a).

**Figure 1:**
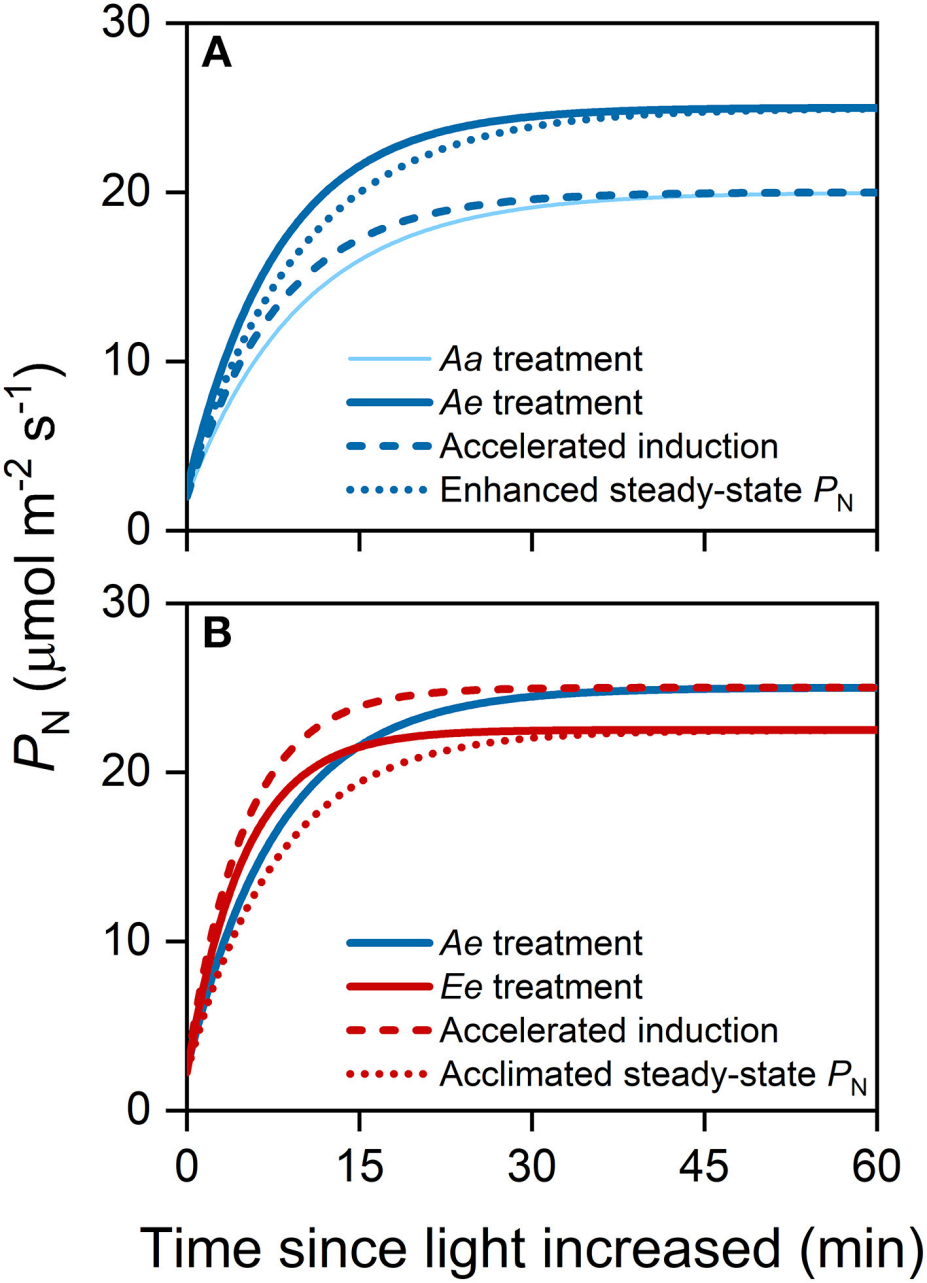
An example illustrating the substrate effect of elevated carbon dioxide (eCO_2_) **(A)** and the acclimation effect of eCO_2_ **(B)** on accumulative carbon gain (ACG) *via* changes in steady-state photosynthetic rates (*P*_N_) and photosynthetic induction. In this study, we assume that the acclimation to long-term eCO_2_ accelerates photosynthetic induction but decreases the steady-state *P*_N_ at high light intensity. All lines were modeled with an exponential function. **(A)** The thin solid curve represents the time course of *P*_N_ following an increase in light intensity from 100 to 1,500 μmol photons m^−2^ s^−1^ under the *Aa* treatment, while the thick solid curve represents that under *Ae* treatment. The dashed blue curve represents the time course of *P*_N_ considering accelerated photosynthetic induction only, i.e., the rate of photosynthetic induction is the same as that under the *Ae* treatment. The dotted blue curve represents the time course of *P*_N_ considering changes in steady-state *P*_N_ only i.e., steady-state *P*_N_ are the same as that under the *Ae* treatment. **(B)** The solid red curve represents the time course of *P*_N_ under the *Ee* treatment. The dashed red curve represents the time course of *P*_N_ considering changes in photosynthetic induction only, i.e., the rate of photosynthetic induction is the same as that under the *Ee* treatment. The dotted red curve represents the time course of *P*_N_ considering changes in steady-state *P*_N_ only i.e., the final, steady-state *P*_N_ is the same as that under the *Ee* treatment.

Accumulative carbon gain (ACG) during a period of time was calculated as


(6) $$ACG(t)=\int_{t_{0}}^{t}P_{N}\left(t\right)dt-P_{N,100}*(t-t_{0})\;$$

where *t*_0_ is the time when light intensity was increased. The eCO_2_ may affect the steady-state *P*_N_ and photosynthetic induction, both of which result in changes in transient *P*_N_ during photosynthetic induction and, thus, affect ACG (Figure 1). To distinguish the relative contribution of eCO_2_ to the ACG *via* accelerated photosynthetic induction from the enhanced steady-state *P*_N_, we decomposed ACG into ideal carbon gain (ICG) and induction efficiency (IE). The ICG during the same period of time was defined as


(7) $$ICG(t)=(P_{N,1500}-P_{N,100})*(t-t_{0})$$

As Equation (7) shows, ICG is determined by steady-state *P*_N_ only. Thus, IE is calculated by dividing the ACG by the ICG, after Tang et al. (1994):


(8) $$IE(t)=\frac{\int_{t_{0}}^{t}P_{N}\left(t\right)dt-P_{N,100}*(t-t_{0})\;}{{\left(P_{N,1500}-P_{N,100}\right)}^{*}(t-t_{0})}$$

Induction efficiency is linearly correlated with *T*_*P*__90%_ (Supplementary Figure 2); thus, the accelerated photosynthetic induction will result in a higher IE. The relative contribution of eCO_2_ on ACG *via* accelerated photosynthetic induction is represented as the percentage difference in ACG under the *Aa* treatment and estimated using IE under the *Ae* and *Ee* treatments.

Additionally, post-illumination carbon gain (PICG) due to the simulated lightfleck was calculated as,


(9) $$PICG=\int_{t_{1}}^{t}\left[P_{N}\left(t\right)-P_{N,50}\right]dt,\;when\;P_{N}\left(t\right)>P_{N,50}$$

where *t*_1_ is the time when light intensity was decreased.

### Statistical Analysis

A two-way ANOVA was performed to evaluate the contribution of the acclimation and substrate effects and their interaction. When the requirement of the normality and homogeneity of variances were met, the differences in the means of different treatments (*Aa, Ae, Ea*, and *Ee*) were then assessed by a least significant difference test. All statistical analyses were carried out with SPSS Statistics Version 26 for Windows (IBM Corp., Armonk, NY, USA) at a significance level of 0.05.

## Results

### Steady-State Photosynthesis Under Different CO_2_ Treatments

In both species, the *P*_N, 100_ and *P*_N,1,500_ in leaves from both growth [CO_2_] treatments were higher when the measurements were conducted under eCO_2_ (Table 2). The averaged *P*_N,1,500_ under the *Ee* treatment was significantly higher than that under the *Aa* treatment by 30.5% in rice and 37.8% in wheat. The averaged *P*_N,50_ did not differ significantly across treatments in rice. In wheat, *P*_N,50_ was significantly lower under the *Ea* treatment compared with the other three treatments. In both species, *g*_s,1,500_ did not differ significantly across treatments (Table 2). In rice, *g*_s,100_ did not differ significantly across treatments, however, *g*_s,50_ was higher under the *Ae* and *Ea* than under the *Ee* and *Aa* treatments. In wheat, both *g*_s,100_ and *g*_s,50_ were higher under the *Aa* and *Ee* than under the *Ae* and *Ea* treatments. The averaged *C*_i,50_, *C*_i,100_, and *C*_i,1,500_ were higher when measurements were conducted under eCO_2_ than under ambient CO_2_ in both species.

**Table 2:** Steady-state photosynthetic rate (*P*_N_), stomatal conductance (*g*_s_), and intercellular CO_2_ concentration (*C*_i_) reached under different light intensities in rice and wheat.

**Parameter**	**CO**_**2**_ **treatment**			
	***Aa***	***Ae***	***Ea***	***Ee***
**Rice**				
*P*_N, 100_	2.22 ± 0.38a	3.77 ± 0.38b	3.08 ± 0.40ab	3.57 ± 0.17b
*g*_s, 100_ ^†^	0.058 ± 0.006	0.094 ± 0.012	0.104 ± 0.021	0.080 ± 0.010
*C*_i, 100_	323 ± 4a	506 ± 17b	335 ± 6a	502 ± 10b
*P*_N, 1, 500_	18.98 ± 0.73a	28.04 ± 1.59b	18.45 ± 0.61a	24.77 ± 1.01b
*g*_s, 1, 500_ ^†^	0.393 ± 0.040	0.430 ± 0.022	0.551 ± 0.057	0.361 ± 0.030
*C*_i, 1, 500_	272 ± 10a	424 ± 8b	299 ± 5c	423 ± 6b
*P*_N, 50_	0.21 ± 0.10	0.84 ± 0.18	1.09 ± 0.34	0.59 ± 0.10
*g*_s, 50_	0.036 ± 0.003a	0.082 ± 0.006b	0.069 ± 0.016bc	0.050 ± 0.004ac
*C*_i, 50_	378 ± 4a	564 ± 2b	363 ± 5c	560 ± 4b
**Wheat**				
*P*_N, 100_	4.41 ± 0.21a	4.78 ± 0.30a	3.46 ± 0.21b	5.07 ± 0.13a
*g*_s, 100_	0.268 ± 0.027a	0.130 ± 0.014b	0.175 ± 0.019bc	0.199 ± 0.024c
*C*_i, 100_	357 ± 3a	518 ± 8b	353 ± 3a	536 ± 5c
*P*_N, 1, 500_	28.24 ± 0.65a	37.65 ± 1.05b	26.70 ± 1.34a	38.91 ± 1.39b
*g*_s, 1, 500_ ^‡^	0.643 ± 0.043	0.503 ± 0.037	0.547 ± 0.047	0.523 ± 0.019
*C*_i, 1, 500_	275 ± 4a	406 ± 6b	270 ± 1a	406 ± 4b
*P*_N, 50_	1.58 ± 0.15a	1.39 ± 0.15a	0.62 ± 0.13b	1.51 ± 0.07a
*g*_s, 50_ ^†^	0.427 ± 0.043a	0.195 ± 0.038b	0.249 ± 0.019ab	0.256 ± 0.011ab
*C*_i, 50_	382 ± 1a	571 ± 1b	385 ± 1a	574 ± 1b

*Values are the means (± SE) of three to four biological replicates, i.e., individual plants for each species. Different letters following the means indicate significant (P < 0.05) differences across treatments within each species*.

*P_N, 50_, P_N, 100_, P_N, 1, 500_, g_s, 50_, g_s, 100_, g_s, 1, 500_, C_i, 50_, C_i, 100_, and C_i, 1, 500_ were steady-state photosynthetic rate (unit μmol CO_2_ m^−2^ s^−1^), stomatal conductance for H_2_O (unit mol H_2_O m^−2^ s^−1^), and intercellular CO_2_ concentration (unit μmol CO_2_ mol^−1^ air) reached at 50, 100, and 1,500 μmol photons m^−2^ s^−1^, respectively, calculated by averaging single values over the last half-minute of each period. The uppercase letters A and E indicate ambient and elevated growth [CO_2_], respectively, and the lowercase letters a and e indicate ambient and elevated measurement [CO_2_] respectively*.

† *Statistical analysis using a Dunnett's T3 test*.

‡ *Statistical analysis using a Kruskal–Wallis test*.

In rice, the differences in *P*_N,100_ and *P*_N,1,500_ were attributable to measurement [CO_2_] only, whereas the differences in *g*_s,100_ and *g*_s,1,500_ were attributable to the interaction of growth and measurement [CO_2_] only. The differences in *g*_s,1,500_ were also, to a less extent, attributable to measurement [CO_2_]. In wheat, the differences in *P*_N_ and *g*_s_ were attributable to measurement [CO_2_]; the differences in *P*_N, 100_ and *g*_s, 100_ were also attributable to the interaction of growth and measurement [CO_2_].

### Dynamic Photosynthesis Under Different CO_2_ Treatments

**Figure 2:**
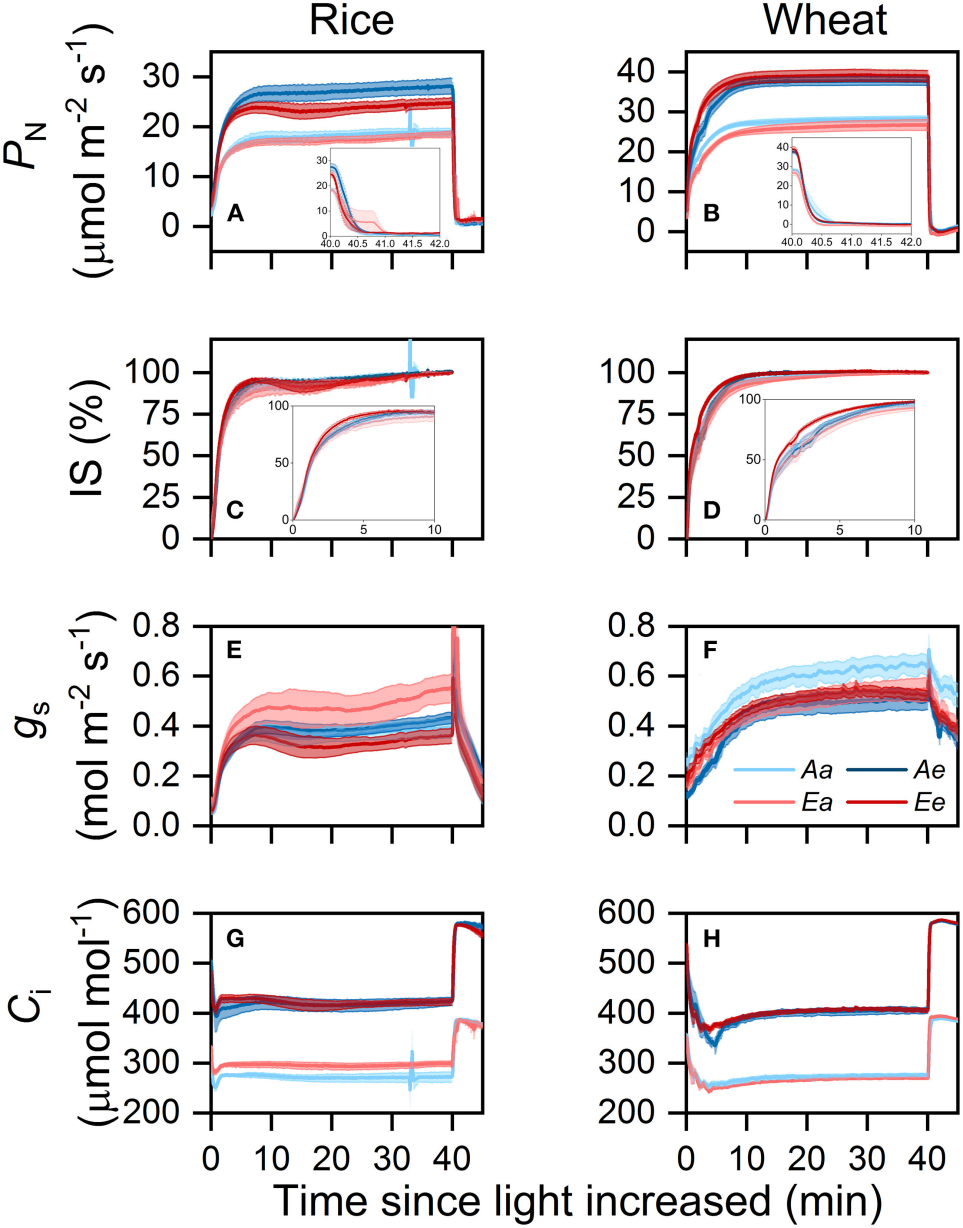
Time courses of *P*_N_ **(A,B)**, IS **(C,D)**, *g*_s_ **(E,F)**, and *C*_i_ **(G,H)** in rice **(A,C,E,G)** and wheat **(B,D,F,H)** leaves following an increase in light intensity from 100 to 1,500 μmol photons m^−2^ s^−1^ and then a decrease in light intensity from 1,500 to 50 μmol photons m^−2^ s^−1^. Values are presented as the means ± SE of three to four biological replicates, i.e., individual plants for each species. *P*_N_, photosynthetic rate; IS, induction state; *g*_s_, stomatal conductance; *C*_i_, intercellular CO_2_ concentration.

In both species, *P*_N_ increased faster when measurements were conducted under eCO_2_ after the sudden increase in light intensity (Figures 2A,B). During the photosynthetic induction and post-illumination period, transient *P*_N_ was higher under the *Ae* than under the *Ee* treatment in rice but was higher under the *Ee* than under the *Ae* treatment in wheat. Transient IS was highest under the *Ee* treatment and lowest under the *Ea* treatment in both species (Figures 2C,D). In rice, the IS was similar between the *Aa* and *Ae* treatments, whereas, in wheat, the IS was surprisingly higher under the *Aa* than under the *Ae* treatment over the first 5 min of photosynthetic induction. In rice, transient *g*_s_ was higher under the *Ae* and *Ea* than under the *Aa* and *Ee* treatments (Figure 2E). In wheat, transient *g*_s_ was higher under the *Aa* than under the *Ae* treatment but was similar between the *Ea* and *Ee* treatments (Figure 2F). During the first 10 min of photosynthetic induction, transient *C*_i_ was higher under the *Ee* than under the *Ae* treatment in both species (Figures 2G,H). Transient *C*_i_ was higher under the *Ea* than under the *Aa* treatment in rice but was higher under the *Aa* than under the *Ea* treatment in wheat. During the post-illumination period, transient *C*_i_ was similar across treatments in both species.

**Figure 3:**
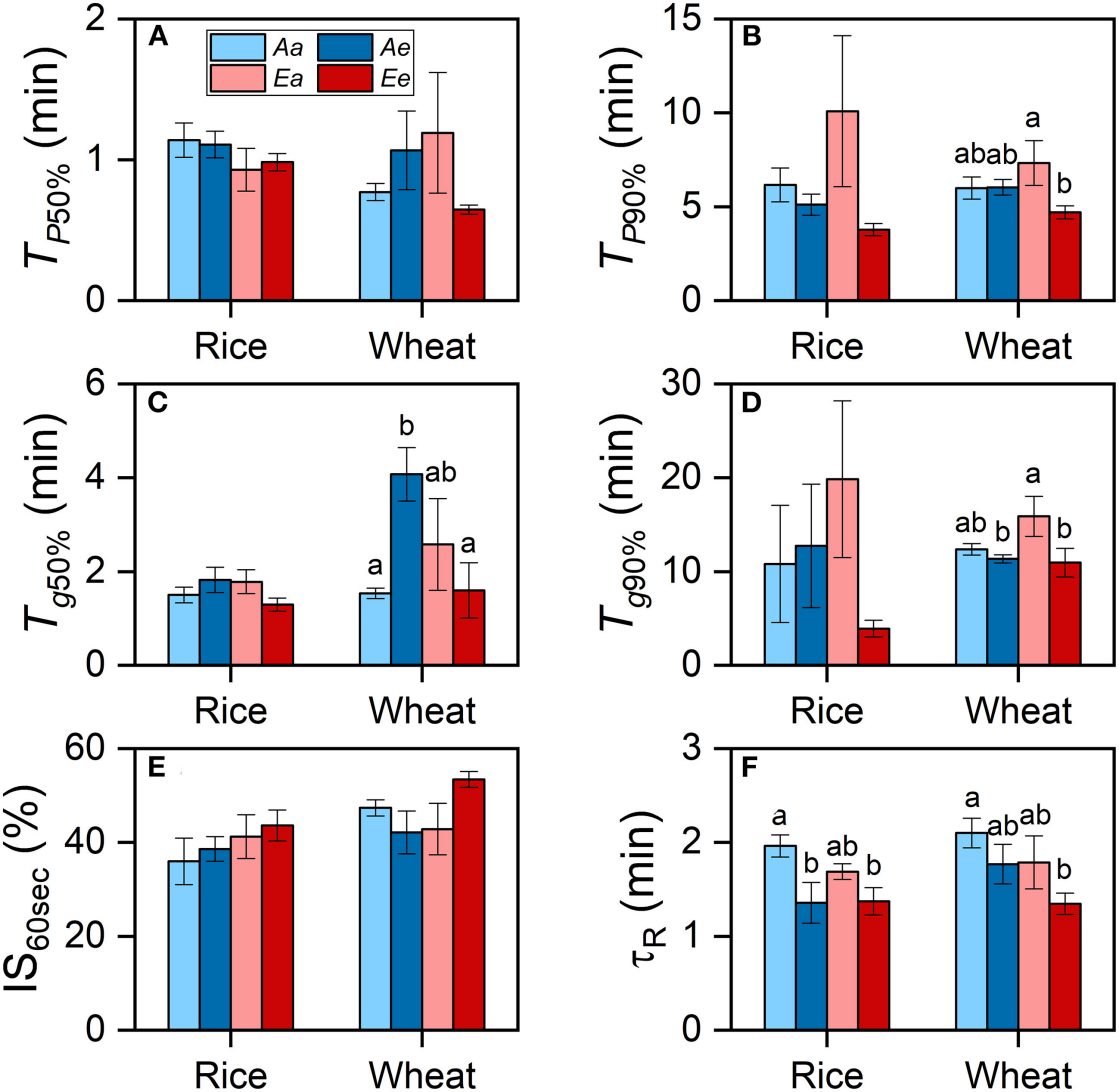
The rates of photosynthetic induction and stomatal opening in rice and wheat leaves. **(A)** Time required for the photosynthetic rate to reach 50% of *P*_N,1,500_ (*T*_*P*__50%_). **(B)** Time required for the photosynthetic rate to reach 90% of *P*_N,1,500_ (*T*_*P*__90%_). **(C)** Time required for stomatal conductance to reach 50% of *g*_s,1,500_ (*T*_g50%_). **(D)** Time required for stomatal conductance to reach 90% of *g*_s,1,500_ (*T*_g90%_). **(E)** The IS reached 30 s following an increase in light intensity (IS_60s_). **(F)** The apparent time constant of Rubisco activation (τ_R_). Bars and vertical lines indicate the means and standard errors of three to four biological replicates, i.e., individual plants for each species, respectively. Different letters above error bars indicate significant differences between two treatments within each species. The absence of letters denotes the absence of significant difference.

In both species, the averaged *T*_*P*__50%_ and *T*_*P*__90%_ were lower under the *Ee* than under the *Aa* and *Ae* treatments, despite that such differences were insignificant (Figures 3A,B). In wheat plants grown at an ambient CO_2_ concentration, *T*_*g*__50%_ was significantly higher under the *Ae* than under the *Aa* treatment, whereas *T*_*g*__90%_ was higher under the *Aa* than the *Ae* treatment (Figures 3C,D). In wheat plants grown at an eCO_2_ concentration, both *T*_*g*__50%_ and *T*_*g*__90%_ were higher under the *Ea* than under the *Ee* treatment. However, *T*_*g*__50%_ and *T*_*g*__90%_ were similar in rice leaves from different growth conditions [CO_2_] or between different measurements [CO_2_] from the same growth [CO_2_]. No significant differences in the IS_60s_ values across treatments were found in both species (Figure 3E). In both species, τ_R_ was shorter when measurements were conducted under eCO_2_ (Figure 3F). In wheat, the differences in *T*_*P*__90%_ were attributable to measurement [CO_2_] and the interaction of growth and measurement [CO_2_] (Table 3). The differences in *T*_*g*__50%_ in both species were attributable to the interaction of growth and measurement [CO_2_] only. In both species, the diffusional limitation was similar between the *Aa* and the *Ea* treatment, i.e., the limitation showed minimal significant differences in leaves from different growth conditions [CO_2_] when measurements were conducted under ambient CO_2_. However, the diffusional limitation was lower under the *Ee* than under the *Ae* treatment. The biochemical limitation tended to be lower when measurements were conducted under eCO_2_, regardless of growth [CO_2_] (Supplementary Figure 3). The percentage of diffusional limitation among the total photosynthetic limitation was lower than 10% during most of the time of the photosynthetic induction period (Supplementary Figures 3A,B). Biochemical limitation dominated over diffusional limitation for the first 3 min of induction in both species.

**Table 3:** The influences of growth (*G*) and measurement CO_2_ concentration (*M*) on the differences in the photosynthetic characteristics of rice and wheat.

**Parameter**	**Factors**		
	**Growth [CO_**2**_]**	**Measurement [CO_**2**_]**	***G* × *M***
**Rice**			
*P*_N, 50_	2.838	0.110	8.912^**^
*P*_N, 100_	1.249	12.005^**^	3.232
*P*_N, 1, 500_	3.693	76.750^***^	1.432
*g*_s, 50_	0.000	2.531	15.160^***^
*g*_s, 100_	1.607	0.229	5.953^*^
*g*_s, 1, 500_	1.727	4.973^*^	11.119^**^
*T_*P*50%_*	2.970	0.015	0.190
*T_*P*90%_*	0.457	3.689	1.874
*T_*g*50%_*	0.399	0.193	4.363^*^
*T_*g*90%_*	0.000	1.700	2.759
IS_60s_	2.350	0.564	0.001
τ_R_	0.606	7.724	0.770
IE_30min_	0.546	1.095	0.395
ACG_30min_	5.374^*^	43.884^***^	0.343
PICG	1.129	2.990	0.027
**Wheat**			
*P*_N, 50_	14.087^***^	9.541^**^	22.845^***^
*P*_N, 100_	2.882	26.314^***^	10.332^**^
*P*_N, 1, 500_	0.020	118.795^***^	1.977
*g*_s, 50_	4.752^*^	17.685^***^	20.164^***^
*g*_s, 100_	0.374	9.378^**^	18.529^***^
*g*_s, 1, 500_	1.360	6.140^*^	3.067
*T_*P*50%_*	0.000	0.313	3.552
*T_*P*90%_*	0.000	4.300^*^	4.582^*^
*T_*g*50%_*	1.656	1.979	10.051^**^
*T_*g*90%_*	1.753	6.336^*^	2.765
IS_60s_	1.070	0.672	5.868^*^
τ_R_	3.825	4.224	0.083
IE_30min_	0.036	8.278^**^	4.200^*^
ACG_30min_	0.121	132.070^***^	2.373
PICG	2.054	6.274^*^	1.838

*Shown are Wald χ^2^ statistics followed by significance symbols, which are*

* *P < 0.05*,

** *P < 0.01, and*

*** *P < 0.001, respectively*.

### ACG and IE

**Figure 4:**
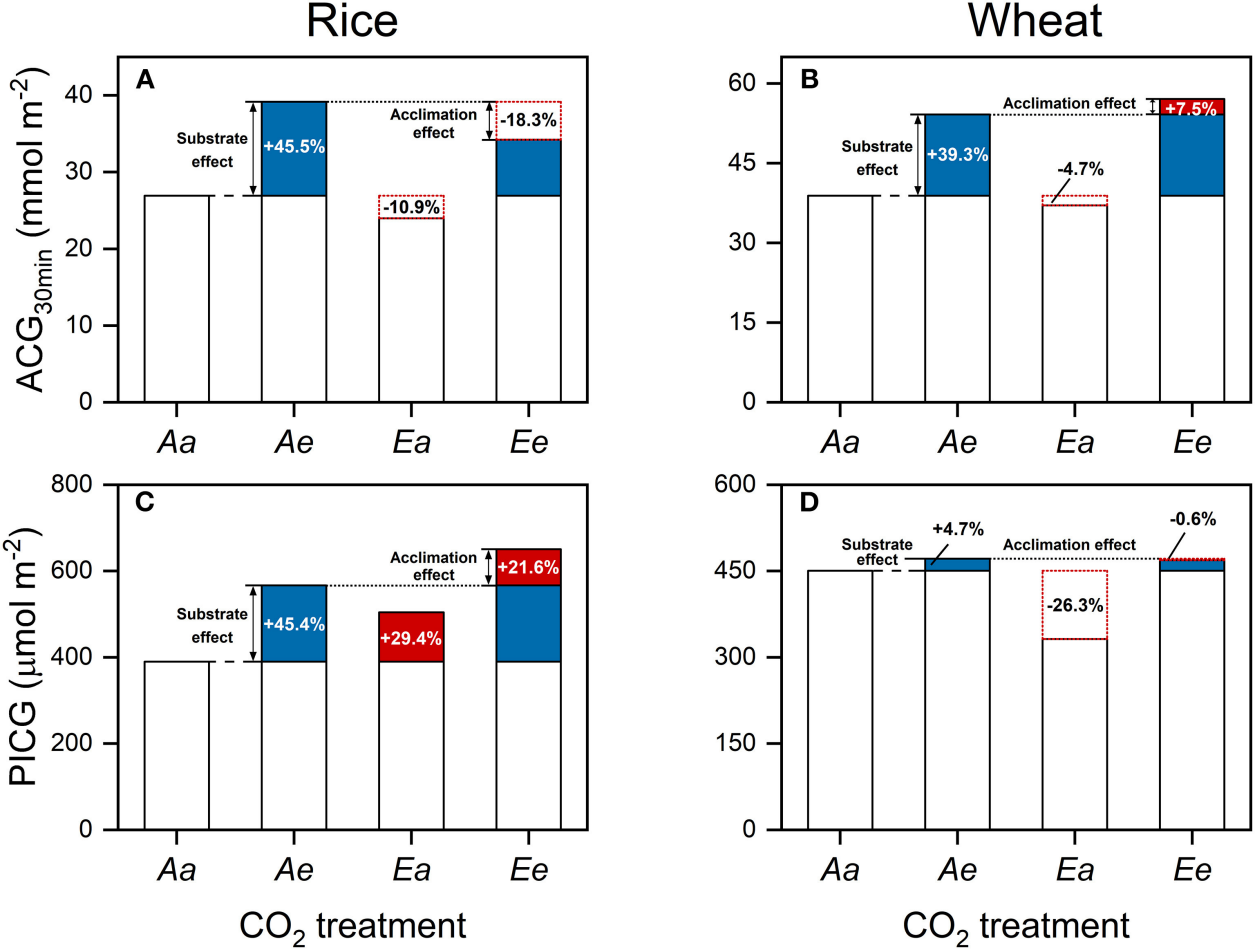
Accumulative carbon gain during a time period of 30 min following an increase in light intensity from 100 to 1,500 μmol photons m^−2^ s^−1^ **(A,B)** and during the post-illumination period **(C,D)** in rice **(A,C)** and wheat **(B,D)** leaves. Values are means of three to four biological replicates, i.e., individual plants for each species. Blue-filled bars lines indicate the substrate effect of eCO_2_, whereas red-filled and open bars with red dotted frames indicate the acclimation effect of eCO_2_. Numbers indicate the extent of the substrate and acclimation effect of eCO_2_ on ACG, taking ACG_30min_ under the *Aa* treatment as the base value.

In comparison with the *Aa* treatment, ACG_30min_ was higher by 45.5 (*Ae*) and 27.2% (*Ee*) in rice (Figure 4A) and by 39.3 (*Ae*) and 46.8% (*Ee*) in wheat (Figure 4B). However, ACG_30min_ was lower under the *Ea* than the *Aa* treatment by 10.9% in rice and by 4.7% in wheat. In comparison with the *Aa* treatment, PICG was higher by 45.4 (*Ae*) and 67% (*Ee*) in rice (Figure 4C) and by 4.7 (*Ae*) and 4.1% (*Ee*) in wheat (Figure 4D). In rice, PICG was higher under the *Ea* than the *Aa* treatment by 29.4% in rice but was lower by 26.3% in wheat.

**Figure 5:**
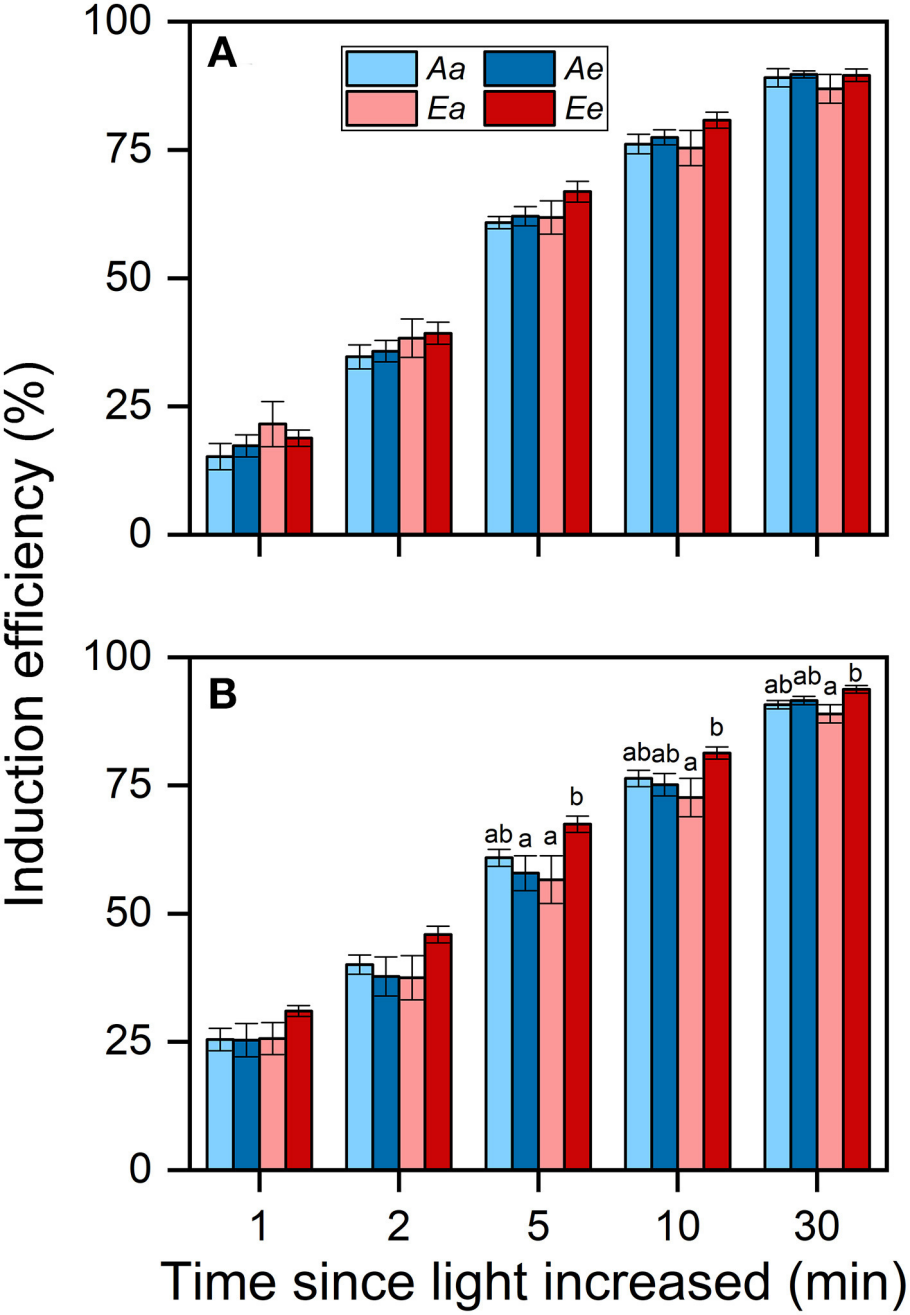
Induction efficiency (IE) in rice **(A)** and wheat **(B)** leaves. Bars and vertical lines indicate the means and SEs of three to four biological replicates, i.e., individual plants for each species, respectively. Different letters above error bars indicate significant differences across treatments within each species. The absence of letters denotes the absence of significant difference.

Despite these changes in ACG_30min_, the IE_30min_ did not differ significantly among the *Aa, Ae*, and *Ee* treatments in both species (Figures 5A,B). In both species, IE_30min_ was lowest under the *Ea* treatment. There were no significant differences observed in IE between the *Aa* and the *Ae* treatments in both species. In wheat, the averaged IE was highest under the *Ee* treatment throughout photosynthetic induction.

**Figure 6:**
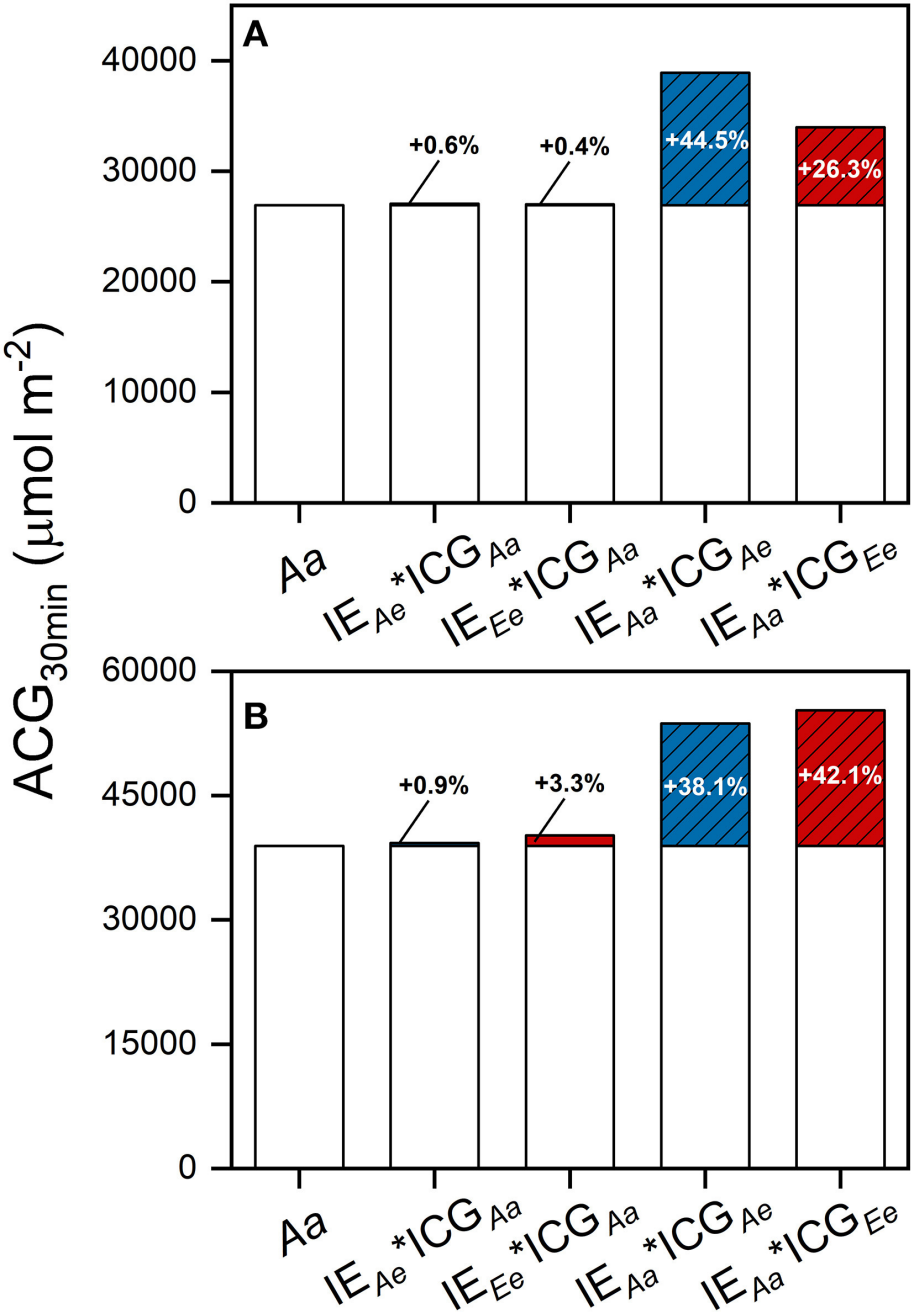
The effects of eCO_2_ on ACG_30min_ by changes in photosynthetic induction and steady-state photosynthesis in rice **(A)** and wheat **(B)** leaves. The ACG under the *Aa* treatment was calculated by integrating *P*_N_ over a time period of 30 min following an increase in light intensity from 100 to 1,500 μmol photons m^−2^ s^−1^. Carbon gains under other hypothetical conditions were calculated by multiplying IE_30min_ and ICG_30min_ measured under different CO_2_ treatments, as the subscripts indicate. IE_30min_, IE over the first 30 min of photosynthetic induction; ICG_30min_, ideal ACG, calculated as the product of (*P*_N, 1, 500_ – *P*_N, 100_) and 30 min. Values are means of three to four biological replicates, i.e., individual plants for each species. Blue bars indicate the substrate effect of eCO_2_ and red bars indicate the sum of the substrate and acclimation effect of eCO_2_. Bars without any patterns indicate the effects of changes in photosynthetic induction and bars with oblique lines indicate the effects of changes in steady-state photosynthesis. Numbers indicate the percentage changes in ACG_30min_, taking ACG_30min_ under the *Aa* treatment as the base value.

To further evaluate the contribution of accelerated photosynthetic induction under the *Ae* and *Ee* treatments to ACG_30min_, we estimated the potential ACG_30min_, assuming no effect of eCO_2_ on steady-state *P*_N_, which was equivalent to no changes in ICG. Compared with the *Aa* treatment, the increase in IE_30min_ under *Ae* alone increased ACG_30min_ by 0.6% in rice and by 0.9% in wheat, whereas the increase in IE_30min_ under *Ee* alone increased ACG_30min_ by 0.4% in rice and by 3.3% in wheat (Figures 6A,B). The changes in steady-state *P*_N_ under *Ae* alone increased ACG_30min_ by 44.5% in rice and 38.1% in wheat, whereas the changes in steady-state photosynthesis under *Ae* alone increased ACG_30min_ by 26.3% in rice and 42.1% in wheat.

### Rubisco Carboxylation Capacity, Stomatal Anatomy, and Element Analysis

The Rubisco carboxylation capacity (*V*_c, max_) was lower at elevated than at ambient growth [CO_2_]; the difference was significant in wheat (Supplementary Table 1). Regardless of growth [CO_2_], *V*_c, max_ was higher in wheat than in rice. There were no significant differences in stomatal density at the two growth conditions [CO_2_] (Supplementary Table 1). Guard cells were shorter at elevated than at ambient growth [CO_2_]; the difference was significant in wheat. No significant differences in carbon or nitrogen content were found between ambient and elevated growth [CO_2_] (Supplementary Table 1).

## Discussion

### Increased CO_2_ Supply Enhances Leaf Dynamic Photosynthesis Without Accelerating Photosynthetic Induction in Crops

Several studies reported accelerated photosynthetic induction in woody species at eCO_2_ (Leakey et al., 2002; Holišova et al., 2012; Tomimatsu and Tang, 2012). In this study, the differences in *T*_*P*_ between the *Aa* and *Ae* treatments were small in two crop species (Figures 3A,B), and the differences in *T*_P50%_ were not related to different measurement [CO_2_]. The modeling analysis also indicates that the changes in IE_30min_ under the *Ae* treatment alone increased ACG_30min_ by <1% in both species (Figure 6). On the contrary, the changes in steady-state *P*_N_ alone increased ACG_30min_ by more than one-third in both species (Figure 6). Acevedo-Siaca et al. (2020) reported that, in rice, increases in the photosynthetic carbon gain in fluctuating light are smaller compared with those in steady state. If the contribution of improved transient *P*_N_ at eCO_2_ on carbon gain were excluded in their study, the increased CO_2_ supply may play a limited role in accelerating photosynthetic induction in crops. Thus, an increased CO_2_ supply improves crop photosynthesis by improving steady-state and transient *P*_N_ (Table 2, Figures 2A,B), rather than accelerating photosynthetic induction.

There are no relevant investigations on why an increased CO_2_ supply imposes limited influences on the rate of photosynthetic induction in crop species, however, we hypothesized that dumbbell-shaped stomata, which respond to light faster than kidney-shaped stomata (Franks and Farquhar, 2007; McAusland et al., 2016; Harrison et al., 2020), lower the diffusion limitation imposed on photosynthetic induction in the crop species. Therefore, crop species may exhibit little changes in the rates of photosynthetic induction when there is a minimal increase in CO_2_ supply. The difference in measurement [CO_2_] in our study (200 μmol CO_2_ mol^−1^ air) was smaller than that in the studies reporting significant effects of eCO_2_ on photosynthetic induction (Table 1).

### Differential Effect of Elevated CO_2_ on Leaf Carbon Gain in Rice and Wheat

The effect of acclimation to eCO_2_ has rarely been distinguished from the effect of increased CO_2_ supply because the measurement [CO_2_] was the same as the growth [CO_2_] in previous studies. In comparison with the *Aa* treatment, the acclimation effect alone resulted in a 18.3% decrease in ACG_30min_ in rice but a 7.5% increase in ACG_30min_ in wheat (Figures 4A,B), suggesting the differential influences of the acclimation effect on the photosynthetic carbon gain between the two crop species. Such differences are likely to be related to the difference in the acclimation effect on *P*_N,1,500_, because the high IE_30min_ under the *Ee* treatment led to a smaller increase in ACG_30min_ than high *P*_N,1,500_, correspondingly ICG_30min_, under the *Ee* treatment in both species (Figure 6). Previous eCO_2_ experiments also show that the enhancement of photosynthesis is greater in wheat than in rice (Long et al., 2006). Wheat allocates less leaf nitrogen to Rubisco and a greater catalytic constant of Rubisco carboxylation than rice (Supplementary Table 1), and thus may have greater benefits from eCO_2_ (Makino, 2011).

In this study, IS_60s_, *T*_*P*__90%_, and IE did not differ significantly between *Ee* and *Ae*, or even between the *Ee* and *Aa* treatments (Figures 2C,D, 3B, 5), suggesting limited influence of acclimation effect on the rate of photosynthetic induction. At timescales of minutes, the rate of photosynthetic induction is mainly determined by the light-activation of Rubisco and stomatal opening (Way and Pearcy, 2012; Kaiser et al., 2015). To speed up the rate of Rubisco activation, more resources should be allocated to Rca, which is necessary for Rubisco activation (Yamori et al., 2012). This requirement is unlikely to be met at eCO_2_, as previous studies reported the decreased content of Rca in rice (Chen et al., 2005) and wheat (Zhang et al., 2009; Aranjuelo et al., 2011). This is consistent with the finding that τ_R_ (Figure 3F) and biochemical limitation (Supplementary Figures 3C,D) did not differ significantly between the *Ae* and *Ee* treatments in both species. On the contrary, the differences in *T*_*P*_ between the *Ae* and *Ee* treatments may be related to the changes in the rate of stomata opening. Accumulating evidence demonstrates that species with fast stomatal opening show fast photosynthetic induction (Drake et al., 2013; Deans et al., 2019; Yamori et al., 2020). In comparison with the *Ae* treatment, the stomata tended to open faster (Figures 3C,D) and stomatal limitation tended to be lower (Supplementary Figures 3A,B) under the *Ee* treatment in both species. We hypothesized that the faster stomatal opening in the leaves acclimated to eCO_2_ was achieved by a decrease in guard cell length (Supplementary Table 1), which is consistent with previous studies (Maherali et al., 2002; Zhu et al., 2018; Zheng et al., 2019). In general, small dumbbell-shaped stomata open faster than large ones within the same genus (McAusland et al., 2016). However, a negative correlation between stomatal size and *T*_*g*__50%_ in the genus *Oryza* was reported recently (Zhang et al., 2019). More research is needed to address how acclimation to eCO_2_ in stomatal morphology affects photosynthesis.

### Implications

The effects of eCO_2_ on the dynamic photosynthesis in crops have been rarely investigated until recently (Acevedo-Siaca et al., 2020; Ohkubo et al., 2020). This study showed that eCO_2_ has limited influences on the rates of photosynthetic induction in two crop species but increased photosynthetic carbon gain greatly by improving steady-state and transient *P*_N_. These findings indicate that photosynthetic carbon loss due to induction limitation may be reduced in the future, under a high CO_2_ world. We acknowledge that the photosynthetic responses to increasing CO_2_ concentration are complex. To project crop photosynthesis and yield in the future, both field experiments and *in silico* modeling, spanning a wide range of CO_2_ concentrations, are urgently needed (Drag et al., 2020). The findings in this study, which clarified the contribution of the substrate and acclimation effects of eCO_2_, imply that dynamic photosynthesis is likely to reduce photosynthetic induction limitation under temporally changing light environments in the future, under a high CO_2_ world.

In comparison with wheat, the photosynthetic acclimation to eCO_2_ in rice compromised the beneficial effect of an increased CO_2_ supply on ACG_30min_ but further improved PICG (Figure 4A). Such an increase in PICG may counterbalance the decrease in ACG if rice leaves receive many brief (<1 min) sunflecks. Nonetheless, photosynthetic characteristics vary greatly among rice and wheat accessions (Qu et al., 2017; Salter et al., 2019; Acevedo-Siaca et al., 2020), and the effects of eCO_2_ on photosynthesis are likely to differ between them. The results presented here do not necessarily suggest an advantageous position for wheat in the future. Instead, this study may provide a potential reason for the lower enhancement of yield in rice than in wheat at eCO_2_ (Long et al., 2006), though detailed assessments are needed because of the large variations in photosynthetic light utilization among them.

## Conclusions

By examining dynamic photosynthesis under four different CO_2_ treatments, this study showed that neither an increased CO_2_ supply nor an acclimation to eCO_2_ imposes large influences on the rates of photosynthetic induction in two crop species. But, an increased CO_2_ supply enhances photosynthetic carbon gain greatly *via* improving steady-state *P*_N_. The acclimation effect of eCO_2_ may compromise, or slightly strengthen, the beneficial effect of an increased CO_2_ supply, depending on the species and the fluctuations in light intensity. Our study suggests that the photosynthetic carbon gain in the two crop species is likely to be enhanced in a CO_2_-enriched future when photosynthetic induction limitation becomes significant for leaf carbon gain.

## Data Availability Statement

The original contributions presented in the study are included in the article/Supplementary Material, further inquiries can be directed to the corresponding author/s.

## Author Contributions

YT and YH conceived and designed the experiment. WS and ZH provided the experimental materials. HK, TZ, YZ, XK, and HS conducted the experiment and collected data. HK, TZ, and YZ analyzed the data and drafted the manuscript. All authors contributed to the editing and revising of the final version of the manuscript.

## Funding

This study was supported by the Key Research of Plant Functional Ecology Program of Peking University (No. 7101302307), National Natural Science Foundation of China (Grant No. 41530533), and Key Laboratory of College of Urban and Environmental Sciences (No. 7100602014).

## Conflict of Interest

The authors declare that the research was conducted in the absence of any commercial or financial relationships that could be construed as a potential conflict of interest.

## Publisher's Note

All claims expressed in this article are solely those of the authors and do not necessarily represent those of their affiliated organizations, or those of the publisher, the editors and the reviewers. Any product that may be evaluated in this article, or claim that may be made by its manufacturer, is not guaranteed or endorsed by the publisher.
