# Supplementary material for: Elevated CO2 Enhances Dynamic Photosynthesis in Rice and Wheat
Source: Front Plant Sci. 2021 Oct 1;12:727374. doi: 10.3389/fpls.2021.727374 (PMC8517259; doi:10.3389/fpls.2021.727374)
Supplement: Supplementary file 1 [file Data_Sheet_1.docx]

**Table S1** Carbon and nitrogen content and stomatal anatomy in rice and wheat leaves grown under ambient (*A*) and elevated (*E*) CO_2_ concentration. Values are the means (± SE) of four to five biological replicates, i.e., individual plants for each species and treatment. Percentage changes are followed by significant significance symbol, which are ^*^ *P* < 0.05 and ^**^ *P* < 0.01.

| Leaf traits | Rice | | | Wheat | | |
| --- | --- | --- | --- | --- | --- | --- |
|  | *A* | *E* | Relative change (%) | *A* | *E* | Relative change (%) |
| *V*_c,max_ (μmol m^−2^ s^−1^) | 80.0 ± 4.1 | 75.3 ± 3.9 | 5.9 | 100.7 ± 3.8 | 87.7 ± 0.9 | 12.9^*^ |
| Carbon content (%) | 42.3 ± 0.5 | 42.4 ± 0.3 | 0.0 | 44.0±0.4 | 44.5±0.1 | +1.2 |
| Nitrogen content (%) | 2.66 ± 0.09 | 2.8 ± 0.10 | -3.0 | 4.6±0.2 | 4.5±0.2 | -2.4 |
| Stomatal density (mm^-2^) | 331 ± 13 | 353 ± 9 | +6.7 | 56±2 | 55±2 | -2.4 |
| Guard cell length (μm) | 23.9 ± 0.3 | 23.2 ± 0.2 | -2.9 | 57.7±0.4 | 54.8±0.3 | -4.9^**^ |


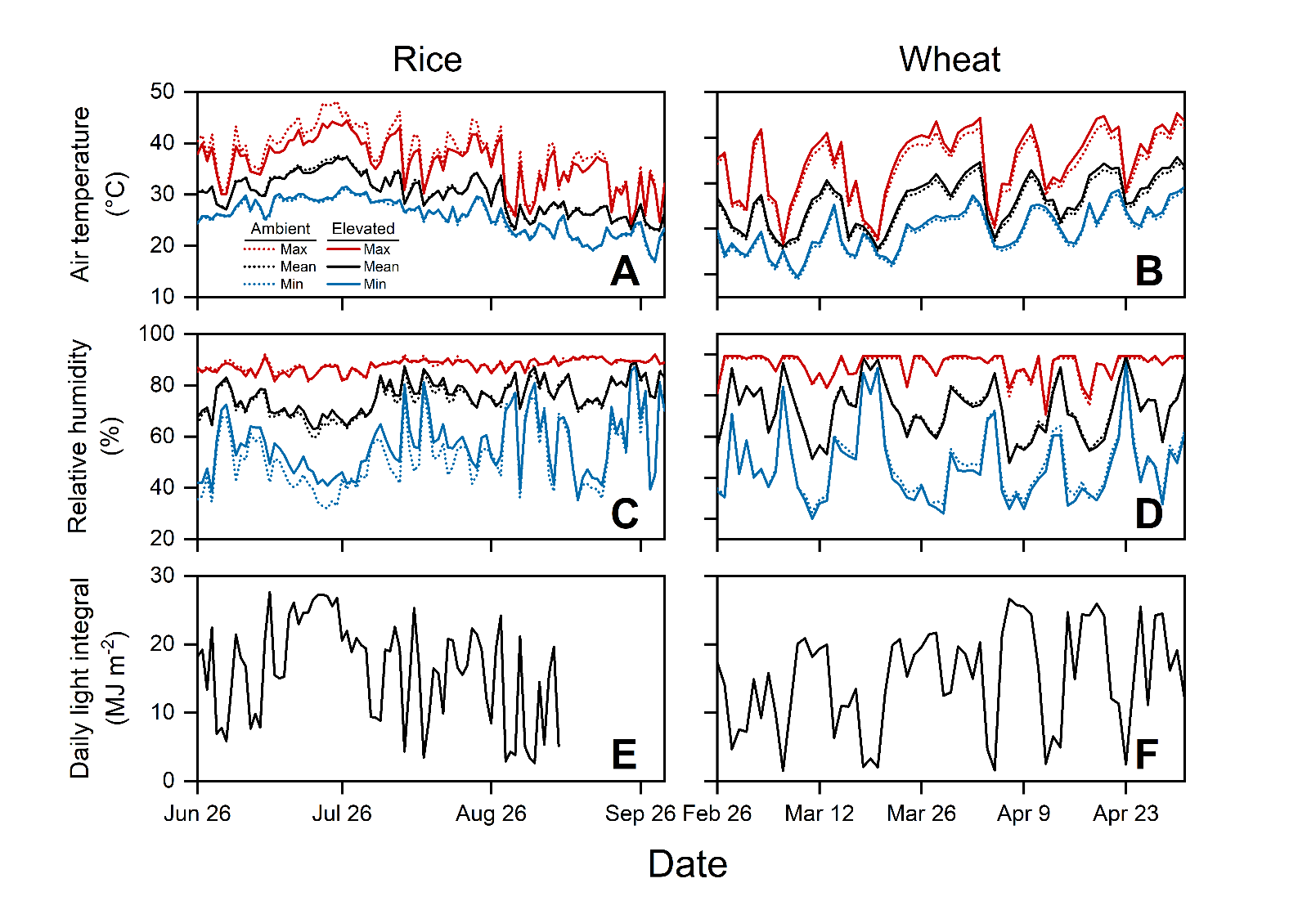


**Figure S1** Meteorological conditions during the growth period of rice (**A**, **C**, **E**) and wheat (**B**, **D**, **F**). (**A**, **B**), Daily maximum, minimum and mean air temperature. (**C**, **D**), Daily maximum, minimum and mean relative humidity. (**E**, **F**), Daily light integral.


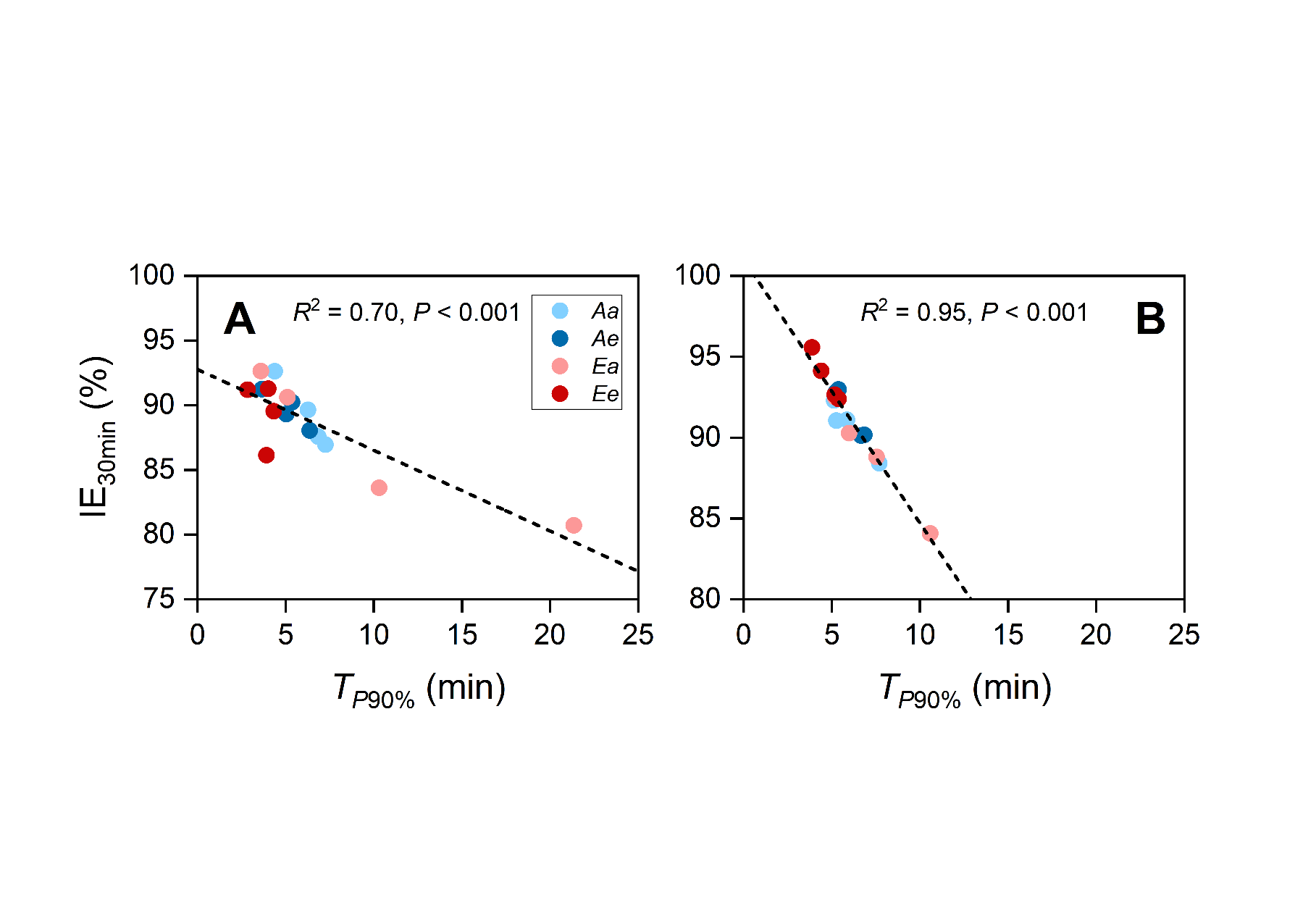


**Figure S2** Induction efficiency over the first 30 min of photosynthetic induction (IE_30min_) as a function of the time required to reach 90% of full induction (*T_P_*_90%_) in rice (**A**) and wheat (**B**). The uppercase letters *A* and *E* to indicate ambient and elevated growth [CO_2_], respectively, and the lowercase letters *a* and *e* to indicate ambient and elevated measurement [CO_2_], respectively.


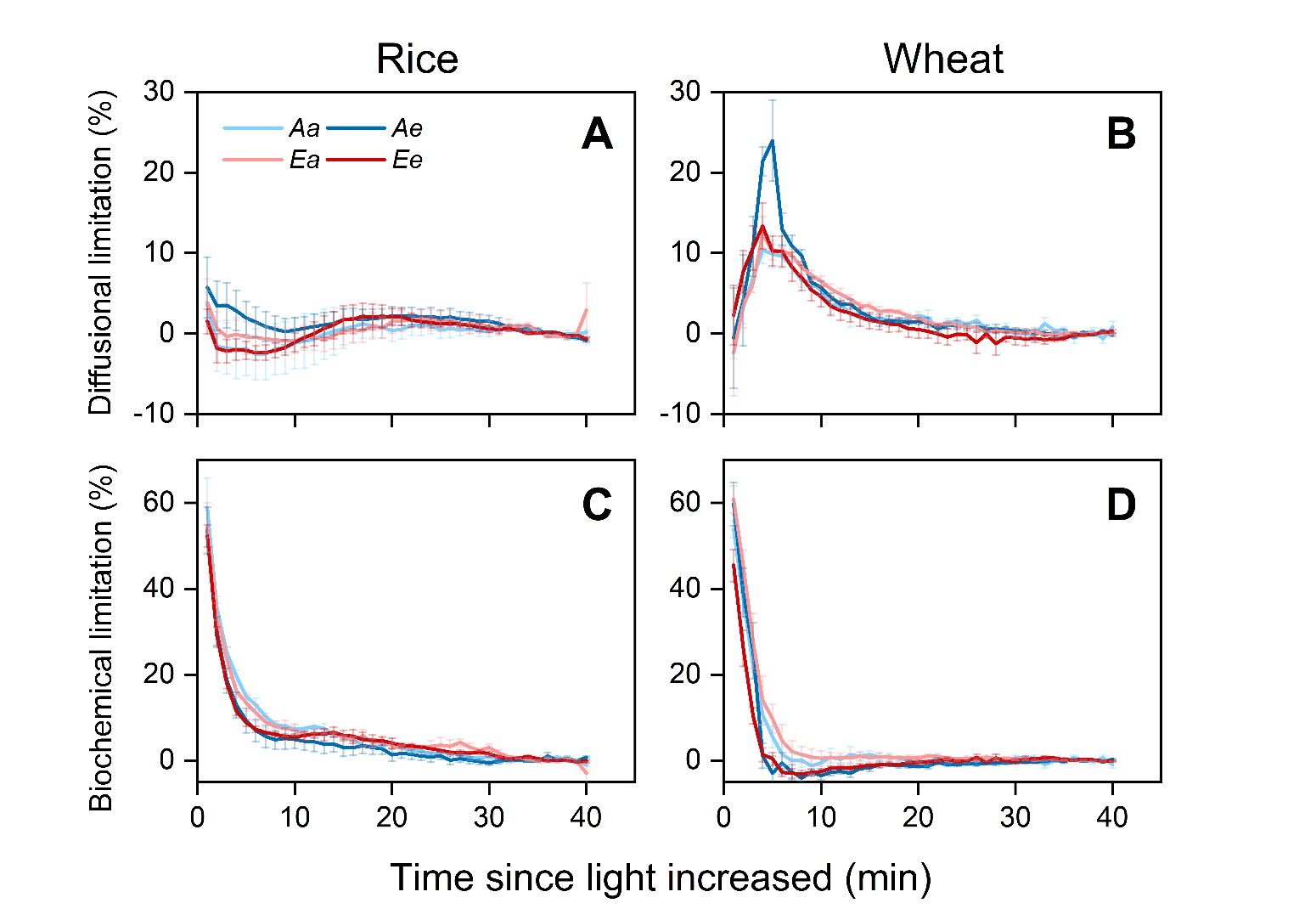


**Figure S3** Time courses of diffusional (**A**, **B**) and biochemical limitation (**C**, **D**) in rice (**A**, **C**) and wheat (**B**, **D**) leaves following an increase in light intensity from 100 to 1500 μmol photons m^-2^ s^-1^. Value are presented as the means ± SE of 3–4 biological replicates, i.e., individual plants for each species.
